# Scanning Mirror Benchmarking Platform Based on Two-Dimensional Position Sensitive Detector and Its Accuracy Analysis

**DOI:** 10.3390/mi16030348

**Published:** 2025-03-19

**Authors:** Hexiang Guo, Junya Wang, Zheng You

**Affiliations:** School of Mechanical Science and Engineering, Huazhong University of Science and Technology, Wuhan 430074, China; m202270773@hust.edu.cn (H.G.); youz@hust.edu.cn (Z.Y.)

**Keywords:** 2D-PSD, accuracy analysis, benchmark test platform, MEMS scanning mirror

## Abstract

A MEMS scanning mirror is a beam scanning device based on MEMS technology, which plays an important role in the fields of Lidar, medical imaging, laser projection display, and so on. The accurate measurement of the scanning mirror index can verify its performance and application scenarios. This paper designed and built a scanning mirror benchmark platform based on a two-dimensional position-sensitive detector (PSD), which can accurately measure the deflection angle, resonance frequency, and angular resolution of the scanning mirror, and described the specific test steps of the scanning mirror parameters, which can meet the two-dimensional measurement. Secondly, this paper analyzed and calculated the angular test uncertainty of the designed test system. After considering the actual optical alignment error and PSD measurement error, when the distance between the PSD and MEMS scanning mirror is 100 mm, the range of mechanical deflection angle that can be measured is (−6.34°, +6.34°). When the mechanical deflection angle of the scanning mirror is 0.01°, the accuracy measured by the test system is 0.00097°, and when the mechanical deflection of the scanning mirror is 6.34°, the accuracy measured by the test system is 0.011°. The test platform has high accuracy and can measure the parameters of the scanning mirror accurately.

## 1. Introduction

Since Peterson designed the first MEMS scanning mirror in 1980 [1], the MEMS scanning mirror has a history of more than 40 years. A large number of scanning mirrors have been designed and developed with four driving modes: electrostatic [2], electromagnetic [3], electrothermal [4], and piezoelectric [5]. It has the advantages of miniaturization and low power consumption and can be used in MEMS Lidar [6], projection display [7], optical coherence tomography [8], optical communication [9], and other scenarios.

The MEMS scanning mirror has three indexes: scanning angle, scanning angle resolution, and resonant frequency. The three indicators of the index test are the internal integrated angle sensor, external calibration, and two test methods. The internal integrated angle sensor is an online measurement; there are piezoresistive, electromagnetic, piezoelectric, capacitive, and photoelectric test modes. The piezoresistive angle sensor has the advantages of a simple process and high integration [10]. It can measure the angle accurately mainly through the Wheatstone bridge and estimate the angle with UKF. The accuracy is about 0.05° [11]. The electromagnetic angular displacement sensor integrates a permanent magnet and a current coil into the MEMS scanning mirror, and the measurement accuracy is ±0.05° [12]. The piezoelectric angle sensor is based on the piezoelectric effect of AlN to measure the angle of the micromirror, and its sensitivity is about 43.96257 mV/° [13]. The photoelectric angle sensor is composed of four photodiodes (PD) and a VCSEL. The deflection angle of the mirror is calculated by measuring the change in the received light energy of the photodiode with an accuracy of 0.0067° [14]. The capacitive angle sensor uses the micromirror as the upper plate and the PCB board as the lower plate and calculates the angle by measuring the capacitance change between the plates with an accuracy of 0.014° [15]. The accuracy of the angle sensor is usually below 0.01°, and the accuracy needs to be calibrated by a more accurate angular reference. The benchmark test method of external calibration can meet the two situations of calibrating the accuracy of the built-in angle sensor of the MEMS scanning mirror and testing the index of the MEMS scanning mirror. Building a high-accuracy scanning mirror benchmark test platform is of great significance for the design and processing of laboratory scanning mirrors and the design and optimization of angle sensors.

For the external calibration scanning mirror index test method, the simplest method is to record the reflection position of the light spot when the MEMS scanning mirror deflects at a certain angle on the coordinate paper and solve the deflection angle of the scanning mirror by the triangulation method, but this method has low accuracy and cannot record the dynamic scanning characteristics of the scanning mirror. In 2016, Faller et al. proposed a mirror position test method based on a Michelson interferometer [16], which calculated the position change distance of a scanning mirror by measuring the change in optical path difference. The optical path of this method is relatively complicated, the measurement procedure is cumbersome, and the back-end algorithm is needed to improve the accuracy. In 2019, Wang Junya et al. proposed a reference angle test method for scanning mirrors based on LDV [11]. This method is to vertically incident the laser spot onto the MEMS scanning mirror and calculate the deflection angle by measuring the change in the vertical position of the laser spot on the mirror. This method requires a relatively accurate coordinate position of the laser spot, although the accuracy is high. However, the measurement angle is relatively small and can only satisfy one-dimensional measurement. In 2004, Valerio Annovazzi-Lodi et al. compared vidicon camera, PSD, and interferometer optical measurement methods for micromechanical mirrors [17]. Many studies have used two-dimensional PSD to test the angle of MEMS scanning mirrors [18,19], but the specific test accuracy of the PSD test method was not described in these papers.

The benchmark test platform can measure the index of the MEMS scanning mirror and test the sensor accuracy of the scanning mirror, which is of great significance for the design and manufacture of the scanning mirror and built-in angle sensor in the laboratory.

Two-dimensional PSD is a photoelectric position detector that uses the surface resistance of a photodiode to convert the spot position on the photosensitive surface into an electrical signal, which can provide continuous position data, high position resolution, and high-speed response and can be used to measure the displacement of the beam center and further calculate the deflection angle of the scanning mirror.

Although there have been many studies on the content of two-dimensional PSD testing MEMS scanning mirrors, and many scholars use two-dimensional PSD to test the angle of MEMS scanning mirrors, there are almost no studies on the accuracy of the two-dimensional PSD testing platform, and no one has made a quantitative description of its test accuracy. In this paper, a benchmarking platform for scanning mirrors based on two-dimensional PSD is built, the error factors affecting the platform are analyzed, and its high accuracy characteristics are verified by quantitative calculation so that it can be used as a benchmark for scanning mirror angle testing and MEMS scanning mirror built-in angle sensor accuracy testing.

This paper designed and built a scanning mirror test benchmark platform based on two-dimensional PSD to realize the testing of the scanning angle, scanning angle resolution, and resonance frequency of the scanning mirror, and the test accuracy of the platform is also analyzed. The test system has high test accuracy and convenient operation and can measure the indicators of two directions at the same time.

## 2. Design and Test Principles

### 2.1. Platform Design and Construction

The test platform mainly includes a two-dimensional PSD (on tark PSM2-45), a MEMS scanning mirror, a laser, a multi-axis displacement table, an AD/DA circuit, and a PC, using the triangulation test principle to test the parameters of the scanning mirror.

The optical path of the test platform is a laser aimed at the center of the MEMS scanning mirror, and the optical path reflected by the scanning mirror is vertically incident to the two-dimensional PSD surface. The two-dimensional PSD, MEMS scanning mirror, and laser are mounted on the multi-axis displacement platform to achieve accurate alignment and error control of the optical path.

**Figure 1:**
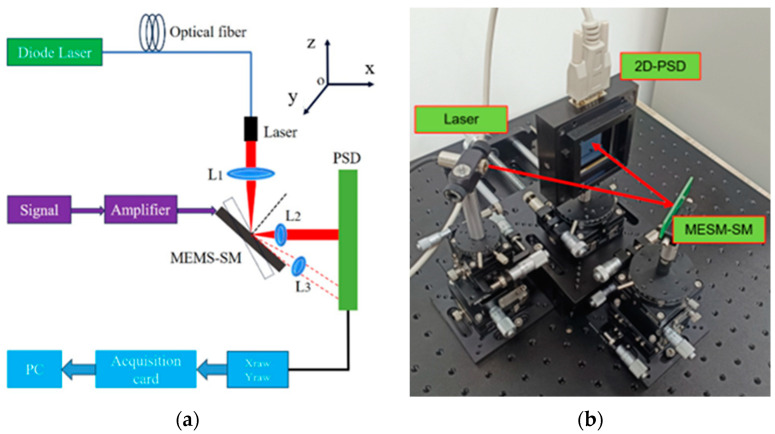
Scanning mirror benchmark test platform based on two-dimensional PSD: (**a**) schematic diagram; (**b**) picture of real products.

All devices are mounted on optical platforms. The laser is installed on the five degrees of freedom displacement platform, with the XYZ rotating around the Z-axis, pitching five degrees of freedom. The model of the three-dimensional displacement table used in the XYZ three degrees of freedom is HLD60-LM-2 of the Hengyang Optical brand, Hongkong, China. The XY-axis stroke is 13 mm, and the accuracy is 0.01 mm. The Z-axis stroke is 10 mm, and the accuracy is 0.02 mm. The model of the rotary table used in the R degree of freedom is HRS60-L of the Hengyang Optical brand. The degree of freedom of rotation about the z-axis is 360° coarse adjustment and ±5° fine adjustment, and the minimum scale is 10′. The model of the pendulum used in the pitch freedom is HWDT60-75 of the Hengyang Optical brand. The stroke of the pitch table is ±10°, and the accuracy of the micrometer used is 0.02 mm. The MEMS scanning mirror is installed on the four degrees of freedom displacement table, with the XYZ rotating around the z-axis with four degrees of freedom. The model of the three-dimensional displacement table used in the XYZ three degrees of freedom is HLD60-LM-2 of the Hengyang Optical brand. The XY-axis stroke is 13 mm, and the accuracy is 0.01 mm. The Z-axis stroke is 10 mm, and the accuracy is 0.02 mm. The model of the electric rotary table rotating around Z is the OMT-23112203DK model of the Hongxing Yang brand, with 360° rotation; the repetitive accuracy of the electric rotary table is ±0.006°, and the minimum step size is 0.0002°. The two-dimensional PSD is installed on the three-degree-of-freedom displacement platform, with three degrees of freedom: Y-axis, rotating around the Z-axis, and pitch. The model of the displacement table used in Y-freedom is HD-8303 of the Hengyang Optical brand. The Y-axis travel is 100 mm, and the accuracy is 0.01 mm. The model of the rotary table used in the R degree of freedom is HRSP60-L of the Hengyang Optical brand. The degree of freedom of rotation about the Z-axis is 360° coarse adjustment and ±5° fine adjustment, and the minimum scale is 5′. The model of the pendulum used in the pitch freedom is HWDT60-75 of the Hengyang Optical brand. The stroke of the pitch table is ±10°, and the accuracy of the micrometer used is 0.02 mm. The schematic diagram and physical diagram of the test platform are shown in Figure 1.

In this test platform, the angle of the reference xoy plane is measured by a level used to regulate the laser’s outgoing beam parallel to the bottom optical platform, with the MEMS scanning mirror and 2D-PSD perpendicular to the optical platform. For degrees of freedom rotating around the Z-axis, a vertical datum is first established on the outer side of the light plate to keep the laser in a constant state. By making parallel lines, the two-dimensional PSD, MEMS scanning mirror, and laser outgoing beam are parallel to the side of the light plate; that is, the two-dimensional PSD is perpendicular to the xoy plane, and the laser outgoing beam is parallel to the xoy plane. Then, the rotating displacement table with the laser and the MEMS scanning mirror is adjusted so that the incidence angle between the incident light and the MEMS scanning mirror is 20°. Due to the accuracy of the turntable and the pendulum, the incidence angle of the laser has a certain error, and the two-dimensional PSD plane is not completely parallel to the datum plane established on the outer side of the light plate, and the related error is the accuracy of the turntable and the pendulum device.

### 2.2. Optical Model of the Test Platform

**Figure 2:**
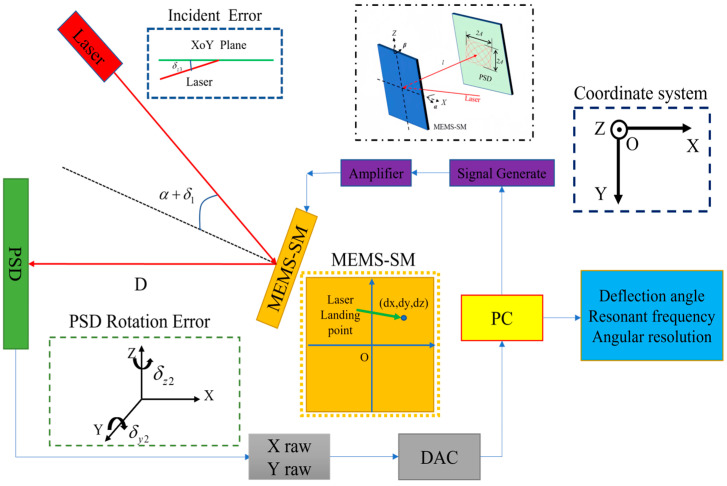
Optical model of the test platform.

Figure 2 shows the global coordinate system of beam propagation from the laser to the PSD and also shows the optical model of beam propagation of the test platform.

The theoretical basis of the derivation of the formulas involved in this chapter is Rodrigue’s rotation formula [20] and the reflection formula of light along the plane [21]. In a coordinate system, the normal vector of a plane and a point on the plane confirm a plane equation.

In the actual scenario, the laser beam does not directly strike the center of the scanning mirror. The error sources in the testing system include angle error when the laser is incident on the scanning mirror; angle error between the laser beam incidence direction and the xoy plane; angle error of 2D-PSD surface rotation about the Z-axis; angle error of two-dimensional PSD surface rotation around the Y-axis; deviation of the laser beam passing through a specific point; and test error in distance measurement between the scanning mirror and PSD.

The rotation axis of the MEMS scanning mirror is set at the origin of the global coordinate system, and when the mirror does not deflect, its angle with the X-axis is α; there is actually an error $\delta_{1}$, so the angle between the scanning mirror and the X-axis is $\left(\pi -\alpha -\delta_{1}\right)$. The normal vector of the scanning mirror plane is as follows:

(1) $$(-\cos(\alpha +\delta_{1}),\sin(\alpha +\delta_{1}),0).$$

The scanning mirror plane passes through the origin; then, the scanning mirror plane equation is as follows:

(2) $$-\cos(\alpha +\delta_{1})x+\sin(\alpha +\delta_{1})y=0.$$

When the scanning mirror rotates a certain angle θ around the Z-axis, its plane normal vector is as follows:

(3) $$(-\cos(\alpha +\delta_{1}-\theta ),\sin(\alpha +\delta_{1}-\theta ),0).$$

The scanning mirror plane passes through the origin; when the scanning mirror rotates a certain angle θ around the Z-axis, the scanning mirror plane equation is as follows:

(4) $$-\cos(\alpha +\delta_{1}-\theta )x+\sin(\alpha +\delta_{1}-\theta )y=0.$$

The distance between the two-dimensional PSD position sensor and the center of the MEMS scanning mirror is D, and the plane of the PSD passing point should be perpendicular to the x-axis and parallel to the y-axis, but in fact, due to the installation error, the surface of the two-dimensional PSD has an angle error $\delta_{z2}$ rotating around the Z-axis and an angle error $\delta_{y2}$ rotating around the y-axis. Through mathematical derivation, the normal vector of the PSD plane is as follows:

(5) $$(\cos\delta_{y2}\cos\delta_{z2},\sin\delta_{z2},-\cos\delta_{z2}\sin\delta_{y2}).$$

The two-dimensional PSD plane passes through the point (−D,0,0); its plane equation is as follows:

(6) $$\cos\delta_{y2}\cos\delta_{z2}(x+D)+\sin\delta_{z2}y-\cos\delta_{z2}\sin\delta_{y2}z=0.$$

The laser is incident to the MEMS scanning mirror, and its incidence angle is $\alpha +\delta_{1}$, which is actually not on the xoy plane; there is an angle error $\delta_{z3}$ in the xoy plane; in reality, the laser beam does not pass through the origin. The laser location coordinates are (dx,dy,dz); the laser beam vector is as follows:

(7) $$(\cos2(\alpha +\delta_{1}),-\sin2(\alpha +\delta_{1}),\sin\delta_{z3}).$$

The linear equation of the laser beam is as follows:

(8) $$\frac{x-\mathrm{dx}}{\cos2(\alpha +\delta_{1})}=\frac{y-dy}{-\sin2(\alpha +\delta_{1})}=\frac{z-\mathrm{dz}}{\sin\delta_{z3}}.$$

When the scanning mirror does not deflect, the reflection vector of the laser beam is as follows:

(9) $$(-1,0,\sin\delta_{z3}).$$

And the linear equation of the reflected laser beam is as follows:

(10) $$\left\{\begin{array}{l} \frac{x-\mathrm{dx}}{-1}=\frac{z-\mathrm{dz}}{\sin\delta_{z3}}\\ y=dy \end{array}\right..$$

When the scanning mirror rotates a certain angle θ around the Z-axis, the coordinates of the intersection point between the laser beam and the scanning mirror plane can be obtained by the linear equation and the plane equation simultaneously, and the formula is as follows:

(11) $$\left\{\begin{array}{l} \frac{x-\mathrm{dx}}{\cos2(\alpha +\delta_{1})}=\frac{y-dy}{-\sin2(\alpha +\delta_{1})}=\frac{z-\mathrm{dz}}{\sin\delta_{z3}}\\ -\cos(\alpha +\delta_{1}-\theta )x+\sin(\alpha +\delta_{1}-\theta )y=0 \end{array}\right..$$

When the scanning mirror rotates at a certain angle θ around the Z-axis, the coordinate of the intersection of the laser beam and the scanning mirror plane is $\left(x_{1},y_{2},z_{1}\right)$; its exact coordinates are accounted for in the subsequent sections.

The reflected laser beam vector is as follows:

(12) $$(-\cos2\theta ,-\sin2\theta ,\sin\delta_{z3}).$$

The linear equation of the inverse laser beam passing through the point $\left(x_{1},y_{2},z_{1}\right)$ is as follows:

(13) $$\frac{x-x_{1}}{-\cos2\theta }=\frac{y-y_{2}}{-\sin2\theta }=\frac{{z-z}_{1}}{\sin\delta_{z3}}.$$

### 2.3. Test Principles

**Figure 3:**
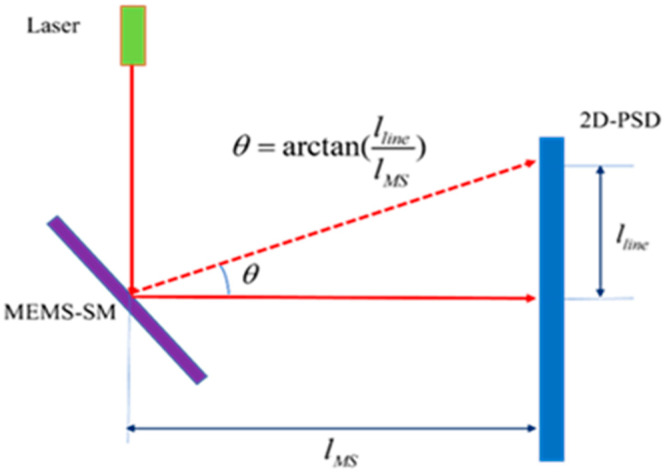
MEMS scanning mirror deflection angle test diagram.

The MEMS scanning mirror mainly has three indexes: scanning angle, scanning angle resolution, and resonant frequency. The three indexes of the scanning mirror are tested by the triangulation method. The test principle diagram is shown in Figure 3.

The deflection angle of the MEMS scanning mirror is different under different driving voltages. Two-dimensional PSD is used to measure the position of the light spot reflected by the scanning mirror under different voltages, calculate the displacement of the reflected light spot, and further calculate the deflection angle. The resonant frequency is tested mainly according to the amplitude of its response under different frequencies. The vibration amplitude is the largest at the resonant frequency. Using this characteristic, the input sinusoidal driving signal frequency of the scanning mirror is changed under a certain voltage, and the vibration amplitude is recorded at different frequencies. The scanning mirror angle accuracy test is measured by the theoretical deflection angle and the actual deflection angle of the scanning mirror. Multiple actual deflection angles are measured, and the standard deviation between the measured value and the theoretical value of the deflection angle is calculated, which is the scanning angle accuracy of the scanning mirror.

(14) $$\theta_{opt}=\arctan(\frac{l_{line}}{l_{MS}}).$$

The above formula is the calculation formula of the deflection optical angle of the scanning mirror. $\theta_{opt}$ is the deflection optical angle of the measured scanning mirror, $l_{line}$ is the moving distance of the outgoing light spot of the scanning mirror, and $l_{MS}$ is the distance between the MEMS scanning mirror and the two-dimensional PSD.

(15) $$\sigma (r)=\sqrt{\frac{1}{N}\sum_{i=1}^{N}{\left(\alpha_{i}-\theta_{i}\right)}^{2}}.$$

The above formula is the calculation formula of the deflection angle accuracy of the scanning mirror. σ(r) is the deflection angle accuracy of the scanning mirror, $\alpha_{i}$ is the theoretical value of the deflection optical angle of the scanning mirror, $\theta_{i}$ is the measured value of the deflection optical angle of the scanning mirror, and N is the number of tests.

## 3. Error Analysis of Test Platform

### 3.1. Measured Angle Calculation

The coordinates of the intersection point between the scanning mirror and the two-dimensional PSD surface can be combined by the linear equation and the plane equation. Since the scanning mirror is deflected around the Z-axis, we just need to find the Y-axis coordinate, and the formula is combined as follows:

(16) $$\left\{\begin{array}{l} \frac{x-\mathrm{dx}}{-1}=\frac{z-\mathrm{dz}}{\sin\delta_{z3}}\\ y=dy\\ \cos\delta_{y2}\cos\delta_{z2}(x+D)+\sin\delta_{z2}y-\cos\delta_{z2}\sin\delta_{y2}z=0 \end{array}\right..$$

When the mirror does not deflect, the Y-coordinate of the intersection point between the reflected laser beam and the two-dimensional PSD surface is as follows:

(17) $$y_{1}=dy.$$

When the scanning mirror rotates at a certain angle θ around the Z-axis, the coordinates of the intersection point between the laser beam and the scanning mirror plane can be obtained by the linear equation and the plane equation simultaneously.

(18) $$\left\{\begin{array}{l} \frac{x-\mathrm{dx}}{\cos2(\alpha +\delta_{1})}=\frac{y-dy}{-\sin2(\alpha +\delta_{1})}=\frac{z-\mathrm{dz}}{\sin\delta_{z3}}\\ -\cos(\alpha +\delta_{1}-\theta )x+\sin(\alpha +\delta_{1}-\theta )y=0 \end{array}\right..$$

The coordinates of the intersection of the laser beam and the scanning mirror plane are as follows:

(19) $$\begin{array}{l} x_{1}=\frac{\sin(\alpha +\delta_{1}-\theta )dy+\sin(\alpha +\delta_{1}-\theta )\tan2(\alpha +\delta_{1})dx}{\sin(\alpha +\delta_{1}-\theta )\tan2(\alpha +\delta_{1})+\cos(\alpha +\delta_{1}-\theta )}\\ y_{2}=\frac{\cos(\alpha +\delta_{1}-\theta )\tan2(\alpha +\delta_{1})dx+\cos(\alpha +\delta_{1}-\theta )dy}{\sin(\alpha +\delta_{1}-\theta )\tan2(\alpha +\delta_{1})+\cos(\alpha +\delta_{1}-\theta )}\\ z_{1}=\frac{\cos(\alpha +\delta_{1}-\theta )\sin\delta_{z3}dx-\sin(\alpha +\delta_{1}-\theta )\sin\delta_{z3}dy}{-\sin(\alpha +\delta_{1}-\theta )\sin2(\alpha +\delta_{1})-\cos(\alpha +\delta_{1}-\theta )\cos2(\alpha +\delta_{1})}+d \end{array}.$$

The coordinates of the intersection point between the reflected laser beam and the two-dimensional PSD surface can be combined by the linear equation and the plane equation. Since the scanning mirror is deflected around the Z-axis, we just need to find the Y-axis coordinate, and the formula is as follows:

(20) $$\left\{\begin{array}{l} \frac{{x-x}_{1}}{-\cos2\theta }=\frac{y-y_{2}}{-\sin2\theta }=\frac{{z-z}_{1}}{\sin\delta_{z3}}\\ \cos\delta_{y2}\cos\delta_{z2}(x+D)+\sin\delta_{z2}y-\cos\delta_{z2}\sin\delta_{y2}z=0 \end{array}\right.$$

The y coordinate of the intersection point between the reflected laser beam and the two-dimensional PSD surface is as follows:

(21) $$\begin{array}{l} y_{3}=\\ \frac{y_{2}(\frac{\cos\delta_{y2}\cos\delta_{z2}}{\tan2\theta }+\frac{\sin\delta_{z3}\cos\delta_{z2}\sin\delta_{y2}}{\sin2\theta })-x_{1}\cos\delta_{y2}\cos\delta_{z2}+z_{1}\cos\delta_{z2}\sin\delta_{y2}-D\cos\delta_{y2}\cos\delta_{z2}}{\frac{\cos\delta_{y2}\cos\delta_{z2}}{\tan2\theta }+\sin\delta_{z2}+\frac{\cos\delta_{z2}\sin\delta_{y2}}{\sin2\theta }}. \end{array}$$

The length of the ray formed on the 2D-PSD is as follows:

(22) $$△y=\frac{y_{3}-y_{1}}{\cos\delta_{z2}}.$$

The distance between the center of the scanning mirror and the two-dimensional PSD position sensor is D; the measured deflection angle is as follows:

(23) $$\theta_{1}=\frac{1}{2}\arctan\frac{△y}{D}.$$

### 3.2. Accuracy Analysis

In this test platform, errors in PSD measurement, misalignment of optical devices, and calibration errors can be considered as the main error sources in this test system. Assuming that all error sources are independent, the variance of angle measurement is as follows:

(24) $$\sigma_{p_{\theta }}^{2}=\sigma_{psd}^{2}+\sigma_{opt}^{2},$$

where $\sigma_{psd}$ and $\sigma_{opt}$ represent the standard deviation caused by PSD and optical alignment, respectively.

The 2D-PSD model is the PSM2-45 from ON Tark. The size of PSD in this test system is 45 × 45 mm, and the resolution is 1.25 μm. The measurement principle is shown in Figure 3. The uncertainty formula is as follows:

(25) $$\sigma_{opt}^{2}={\left(\frac{\partial \theta }{\partial l_{\mathrm{line}}}\right)}^{2}*\sigma_{l_{\mathrm{line}}}^{2}+{\left(\frac{\partial \theta }{\partial l_{MS}}\right)}^{2}*\sigma_{l_{MS}}^{2}.$$

In the above formula, $\sigma_{l_{line}}$ is the uncertainty of PSD measurement, and $\sigma_{l_{MS}}$ is the uncertainty of distance measurement between PSD and scanning mirror.

**Figure 4:**
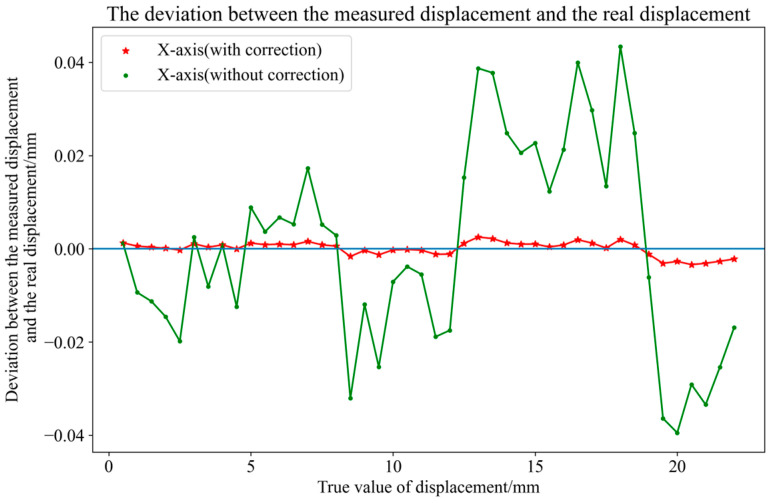
PSD accuracy calibration data.

According to the specification of the 2D-PSD used, the linearity of this PSD is 0.3%. In order to reduce the interference of PSD nonlinear error [22] on the overall experimental platform, the accuracy calibration experiment was first carried out with an electric displacement table. The model of the electric table used was LTS150C of Thorlabs. The accuracy of the electric table was 2 μm, and the minimum step was 100 nm. During the calibration experiment, the laser was vertically incident to the surface of the 2D-PSD. The laser was mounted on the electric displacement platform. The step of the electric displacement platform was 0.5 mm, that is, 500 μm, and it moved from the center of the PSD to the edge of the PSD by 22 mm. The data in the green line in Figure 4 are PSD accuracy calibration data before correction, and the X-axis uncertainty is less than 21.07 μm.

In this paper, a BP neural network is used to correct the nonlinear error of PSD. The BP neural network used in the experiment has four layers, two hidden layers, and a two-input and two-output structure. The input is the displacement coordinate of the actual output of PSD, and the output is the ideal coordinate, and the number of neurons in each hidden layer is 30 and 50.

In Figure 4, the red line data are the data corrected by the BP neural network, and the accuracy of PSD after correction is 1.46 μm.

Considering all errors in optical alignment, the optical uncertainty can be expressed as follows:

(26) $$\begin{array}{l} \sigma_{\theta ,opt}^{2}\\ ={\left(\frac{\partial \theta }{\partial \delta_{y2}}\right)}^{2}\sigma_{\delta y2}^{2}+{\left(\frac{\partial \theta }{\partial \delta_{z2}}\right)}^{2}\sigma_{\delta z2}^{2}+{\left(\frac{\partial \theta }{\partial \delta_{z3}}\right)}^{2}\sigma_{\delta z3}^{2}+{\left(\frac{\partial \theta }{\partial \delta_{1}}\right)}^{2}\sigma_{\delta 1}^{2}.\\ +{\left(\frac{\partial \theta }{\partial \delta_{dx}}\right)}^{2}\sigma_{\delta dx}^{2}+{\left(\frac{\partial \theta }{\partial \delta_{dy}}\right)}^{2}\sigma_{\delta dy}^{2}+{\left(\frac{\partial \theta }{\partial \delta_{dz}}\right)}^{2}\sigma_{\delta dz}^{2}+{\left(\frac{\partial \theta }{\partial \delta_{D}}\right)}^{2}\sigma_{\delta D}^{2} \end{array}$$

**Figure 5:**
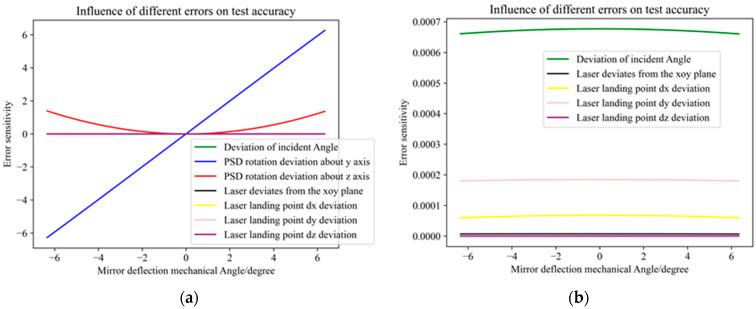
The influence of each error on the accuracy of the test system. (**a**) PSD and other errors; (**b**) other errors excluding PSD errors.

Figure 5 shows the effect of each error on the measured angle.

As can be seen from the Figure 5 above, the rotation angle of PSD around the Y and Z axes has the greatest impact on the optical error, and the other five errors are very small compared with the two errors. The specific impact of the other five errors on the test system is shown in Figure 5b.

### 3.3. Accuracy Calculate

In the specific calculation, the incident angle of the laser to the scanning mirror is set to 20 degrees, the distance D between the PSD and the mirror surface is set to 100 mm, the uncertainty of the measured distance is set to 10 μm, and the size of the PSD is 45 mm × 45 mm. At this time, the maximum optical deflection angle that can be measured is 25.2°. The angle errors of the laser incidence angle to the scanning mirror $\delta_{1}$, the angle error of the laser beam incidence direction with respect to the xoy plane $\delta_{z3}$, the angle error of the two-dimensional PSD surface rotation around the Z-axis $\delta_{z2}$, and the angle error of the two-dimensional PSD surface rotation around the y-axis $\delta_{y2}$ are all set to 0.1°, with an uncertainty of 0.1°. The coordinates of the laser point are dx=dy=dz=20 μm, and the uncertainty is set to 20 μm.

(27) $$\left\{\begin{array}{l} \delta_{1}=\delta_{y2}=\delta_{z2}=\delta_{z3}=0.1^{{}^{\circ}}\\ \sigma_{\delta 1}=\sigma_{\delta y2}=\sigma_{\delta z2}=\sigma_{\delta z3}=0.1^{{}^{\circ}}\\ \sigma_{D}=10\;\mu m\\ D=10\;\mathrm{mm}=100000\;\mu m\\ dx=dy=dz=20\;\mu m\\ \sigma_{dx}=\sigma_{dy}=\sigma_{dz}=20\;\mu m\\ \alpha =20^{{}^{\circ}} \end{array}\right..$$

**Figure 6:**
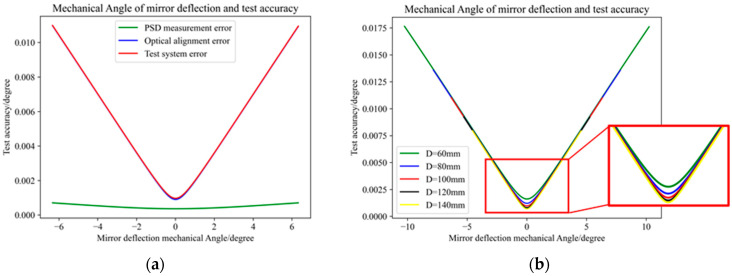
Test platform accuracy and deflection angle relationship: (**a**) D = 100 mm; (**b**) different D.

Figure 6a shows the relationship between the variation in the deflection angle of the scanning mirror and the uncertainty of the test system. The X-axis is the deflection angle of the scanning mirror, and the Y-axis is the uncertainty of the test system. The red line in the figure is the total error of the test system, the blue line is the error generated by the optical alignment, and the green line is the measurement error from the PSD. It can be seen from the above figure that the error of the test system mainly comes from the optical alignment error.

When the distance D between the PSD and the MEMS scanning mirror is different, the maximum measurement range of the scanning angle of the test system is different. As can be seen from Figure 6b, when the deflection angle exceeds a certain range, the different distance D has almost no impact on the uncertainty of the test system. Only when the deflection angle is small does the distance D have a greater impact on the uncertainty of the test system, and the greater the distance D is, the greater the distance D is. The smaller the uncertainty of the system, the smaller the system error.

It can be calculated that when the distance between the PSD and the MEMS scanning mirror is 100 mm, the range of mechanical deflection angle that can be measured is (−6.34°, +6.34°). As can be seen from Figure 6 above, the test accuracy of the scanning angle of the scanning mirror is not unchanged but related to the scanning angle of the scanning mirror. When the scanning angle is small, the test accuracy of the test platform is high, and when the scanning angle is large, the test accuracy of the test platform is reduced. According to the calculation results, when the deflection angle of the scanning mirror is 0.01°, the scanning angle test accuracy of the testing platform is as high as 0.00097°; when the deflection angle of the scanning mirror is 6.34°, the scanning angle test accuracy of the testing platform is 0.011°.

The accuracy of the built-in angle sensor of the scanning mirror is usually lower than 0.01°, and the accuracy of the test platform is higher than that of the built-in angle sensor of the scanning mirror, which can be used as the benchmark of the built-in angle sensor.

### 3.4. Test Platform Error Calibration

**Figure 7:**
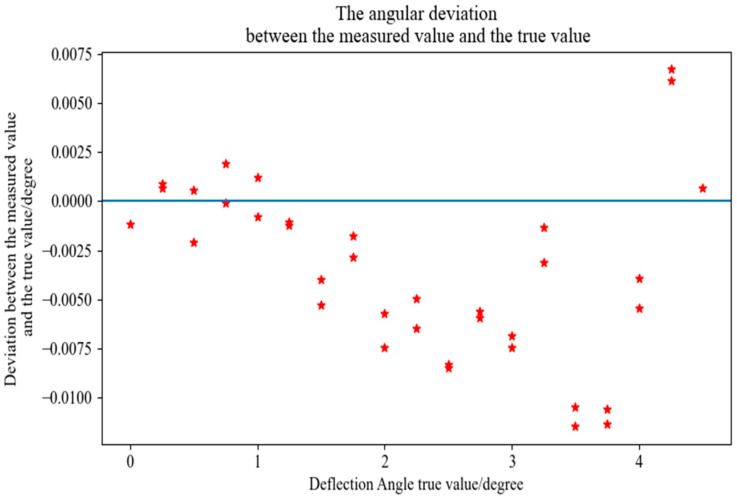
Test platform error calibration data.

According to the error and uncertainty requirements in the above section, a two-dimensional PSD scanning mirror test platform was built, and the calculated results were verified and compared. A customized electric rotating table was used to verify the accuracy of the test system. The minimum step size of the rotating table was 0.0002°, and the repeated positioning accuracy was ±0.0006°. The MEMS scanning mirror is mounted on the electric rotating table, and the electric rotating table is rotated instead of the scanning mirror deflection, and the two-dimensional PSD output data are recorded at a step length of 0.25° in the range of (0, 4.5°). In the experiment, the rotation angle of the electric rotating table is taken as the true value of the angle, and the measurement angle of the two-dimensional PSD is taken as the measurement value. The experimental data are shown in the Figure 7 below. The blue line in the Figure 7 is the baseline error of 0, and the red stars represent the deviation of the true value from the ideal value.

It can be seen from the data in the above Figure 7 that the maximum deviation between the test angle value and the real value is 0.011° and the average deviation value of the measurement is 0.0044°, which is not much different from the actual calculation results. The gap between the two may be due to external environmental vibration or airflow interference, laser spot quality problems, and other factors.

### 3.5. Actual Test Process

In addition to the test platform mentioned above, the hardware ADALM2000 is used for signal output and acquisition, which has a 2-channel 12-bit ADC and 2-channel 12-bit DAC, and the high-voltage amplifier HA-8202A is used to amplify the low-voltage signal output by ADALM2000. The OT-301 Position Sensing Amplifier is used to process the output signal of 2D-PSD. The output voltage range of OT-301 is (−10 V, +10 V), and the spot position range corresponding to the output of 2D-PSD is (−22.5 mm, +22.5 mm). There is a U = k × x relationship between the output voltage and the position of the spot, where k is the proportional coefficient, x is the position coordinate of the spot, and U is the output voltage of OT-301. Through the output voltage signal of OT-301 collected by ADALM2000, the position of the light spot can be calculated, and the moving distance of the light spot can be further calculated. If the distance between the MEMS scanning mirror and the 2D-PSD is known, the deflection angle of the MEMS scanning mirror can be calculated by triangulation.

As for the measurement of the distance D between the MEMS scanning mirror and the 2D-PSD, there are usually two methods.

The first method: After adjusting the optical path of the test platform, the output signal of the 2D-PSD collected by the ADALM2000 is recorded and further converted into the position coordinates of the light spot. Then, the high-accuracy electric displacement table mounted under the MEMS scanning mirror is rotated at a fixed angle, and the position of the light spot mapped on the 2D-PSD changes. The output signal (spot position coordinate) of 2D-PSD collected by ADALM2000 is recorded again, and the distance d of spot movement is calculated. Then, the distance D between the MEMS scanning mirror and the 2D-PSD can be solved by a trigonometric function. The relevant parameters of the electric rotary table are mentioned in Section 2.1.

**Figure 8:**
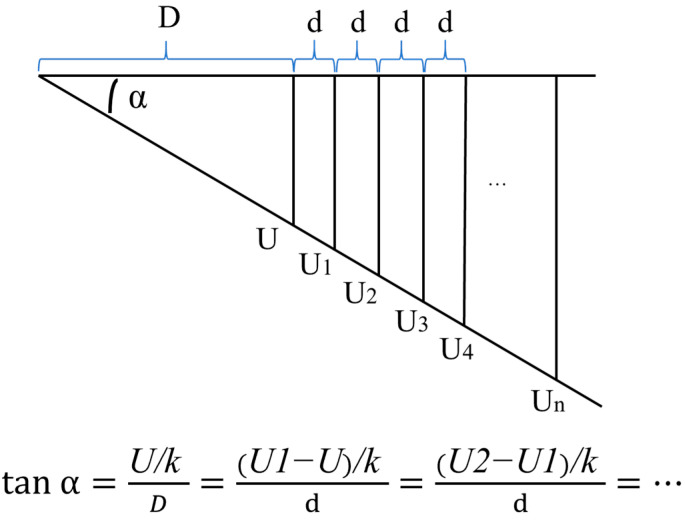
Method of distance calculation between MEMS scanning mirror and 2D-PSD.

The second method: After adjusting the optical path of the test platform, the 2D-PSD output signal collected by the ADALM2000 is recorded and further converted into the position coordinates of the light spot. The electric rotary table mounted under the MEMS scanning mirror rotates at an angle α at will to record the 2D-PSD output signal (spot position coordinates) collected by the ADALM2000. After that, the Y-degree-of-freedom displacement platform equipped with 2D-PSD was moved several times; each time, the moving distance was d, and 2D-PSD output signals (spot position coordinates) collected by ADALM2000 were recorded after the movement. According to the moving distance d and the output signal of the 2D-PSD (spot position coordinates), the distance D between the MEMS scanning mirror and the 2D-PSD can be obtained by solving the average value of the trigonometric function several times. The relevant parameters of the displacement table for Y degrees of freedom are mentioned in Section 2.1. Figure 8 shows the test principle of the above method.

The three indicators of the MEMS scanning mirror can then be measured using the method described in Section 2.3. In order to ensure the accuracy of the test and prevent interference from external factors, the test time is usually extended to ensure that the ADALM 2000 can fully collect the output signal of the 2D-PSD.

## 4. Conclusions

This paper designed and built a scanning mirror test benchmark platform based on two-dimensional PSD, which can test the scanning angle, scanning angle accuracy, and resonant frequency of the scanning mirror. The test system mainly includes a position sensor (PSD) installed on the displacement table, a multi-degree-of-freedom displacement table, and a laser and scanning mirror to be tested. The beam displacement on the PSD is converted to a mirror angle measurement.

In addition, the accuracy of the test platform is analyzed, the optical model of the whole system is established, the functional relationship between the system accuracy and each physical quantity is deduced, and the optical error and PSD measurement error are analyzed in detail. By calculation, when the distance between the scanning mirror and the PSD photosensitive surface is 100 mm, the mechanical deflection angle of ±6.34° can be tested; when the scanning angle of the scanning mirror is 0.01°, the test accuracy is 0.00097°; when the scanning angle of the scanning mirror is 6.34°, the test uncertainty is 0.011°. Overall, compared with the existing MEMS scanning mirror and the angle sensor integrated into the scanning mirror, the test accuracy of the test system is more than ten times and can be used as a benchmark test platform for the scanning mirror, which can efficiently and accurately test the parameters of the scanning mirror.

Finally, this paper verifies the accuracy of the test platform by using an electric rotary displacement table with a repeatability of 0.0012°, which verifies that the test platform has the characteristics of high accuracy, can meet the needs of MEMS scanning mirror index testing, and can be used as a benchmark for the angle sensor built into the MEMS scanning mirror. It provides an important reference value for the design and processing of laboratory MEMS scanning mirrors and the design optimization of built-in angle sensors.

## Data Availability

The original data presented in the study are included in the article.
